# *Lacticaseibacillus rhamnosus* GG Survival and Quality Parameters in Kefir Produced from Kefir Grains and Natural Kefir Starter Culture

**DOI:** 10.3390/foods11040523

**Published:** 2022-02-11

**Authors:** Amin Yousefvand, Xin Huang, Mehdi Zarei, Per Erik Joakim Saris

**Affiliations:** 1 Department of Food Hygiene, Faculty of Veterinary Medicine, Shahid Chamran University of Ahvaz, P.O. Box 6135783-151, Ahvaz 61, Iran; zarei@scu.ac.ir; 2 Department of Microbiology, Faculty of Agriculture and Forestry, University of Helsinki, Viikinkaari 9, P.O. Box 56, FI-00014 Helsinki, Finland; per.saris@helsinki.fi; 3 Department of Food and Nutrition, Faculty of Agriculture and Forestry, University of Helsinki, Agnes Sjöberginkatu 2, P.O. Box 66, FI-00014 Helsinki, Finland; xin.huang@helsinki.fi

**Keywords:** probiotic, kefir, natural kefir starter culture, kefir grain, viscosity

## Abstract

The study aimed to determine the effect of starter cultures (kefir grains and natural kefir starter culture without grains) on *Lacticaseibacillus rhamnosus* GG (LGG) survival and on the quality characteristics of kefir. To this end, the viability of probiotic *L. rhamnosus* GG strain and the rheological properties and quality parameters of kefir beverages were tested during storage over 21 days at 4 °C. The final LGG counts were 7.71 and 7.55 log cfu/mL in natural kefir starter culture and kefir grain, respectively. When prepared with probiotic bacteria, the syneresis values of kefir prepared using natural kefir starter culture was significantly lower (*p* < 0.05) than that of kefir made using grains. However, the viscosity indices, hysteresis loop, and dynamic moduli were similar between kefir made with natural kefir starter culture and other kefir formulations (*p* > 0.05). Moreover, all samples showed shear-thinning behavior. The flavor scores for kefir prepared using natural kefir starter culture were significantly higher than for the other samples (*p* < 0.05), but overall acceptability was similar at the 10-day assessment across both starters (with and without grain) after the addition of probiotic bacteria (*p* > 0.05). Overall, the results indicate that natural kefir starter culture could be a potential probiotic carrier.

## 1. Introduction

Kefir is an acidic-alcoholic fermented dairy beverage with unique attributes such as a slightly sour and yeasty flavor, and a viscous and creamy density. Kefir grains are white to yellowish, irregularly shaped cauliflower-like grains that are traditionally used to manufacture kefir [1,2]. There is a consortium of many bacteria from *Lacticaseibacillus*, *Lactobacillus*, *Lactococcus*, *Leuconostoc*, and *Streptococcus* genera while yeast genera contain *Kluyveromyces*, *Candida*, *Saccharomyces*, and *Pichia* embedded in a resilient water-soluble exopolysaccharide (EPS) matrix, known as kefiran, which produces lactic acid and alcohol, respectively [3,4,5]. The consumption of kefir has many advantages, including alleviation of lactose intolerance symptoms [6], deterrence of pathogenic bacteria [5], as well as anti-inflammatory [7] and antioxidant activity [8]. It has been reported that kefiran in kefir acts as the delivery mechanism of probiotic organisms. Accordingly, kefir has potential to carry probiotic bacteria [9]. It was recently shown that lactic acid bacteria (LAB) counts in kefir were higher from kefir grains than commercial kefir starter culture, suggesting that the commercial starter is not capable of promoting optimal LAB survival [10]. More recently, some commercial kefir starter cultures are existent for industrial production, but they often contain limited yeast species [11]. Due to the lack of reliable population of microorganisms in commercial kefir starter cultures, a remarkable diminution in sensory properties is usually observed. In fact, fermented dairy products develop their unique organoleptic and functional attributes as result from the metabolic activity of the multispecies microflora, where more than one type of interaction may occur concomitantly [12]. Kefir can also be manufactured from natural kefir starter culture, which is initially captured from kefir grains with the same microbial composition. Its use as a starter culture and potential LAB carrier for industrial applications is much easier than kefir grains. 

Currently, the consumption of probiotic-containing fermented dairy products is accepted among the general public. Probiotic bacteria are defined as live microorganisms, which, when administered in adequate amounts, provide health benefits to the host [13]. For example, consumption of probiotic organisms has been associated with the alleviation of lactose intolerance symptoms, improvement of stomach and colon health, modulation of the host immune system, inhibition of pathogenic bacteria, as well as anti-inflammatory, antidiabetic, and anticarcinogenic properties [14,15,16]. Dairy products with probiotic claims should maintain at least 10^7^ cfu/mL of viable cells as a satisfactory level [17].

The majority of probiotic bacteria used in the food industry are LAB, mostly lactobacilli species [18]. Among them, the *Lacticaseibacillus rhamnosus* GG (LGG) strain has been extensively studied. It has been established as a non-spore-forming, non-motile, facultative heterofermentative, anaerobic, and catalase-negative bacterium [19,20]. Moreover, some studies have demonstrated that the probiotic LGG strain can persist in an acidic environment, for example while passing through the gastrointestinal tract, which represents a harsh environment for probiotics [15,21].

Many health benefits have been attributed to the LGG strain, including preventing and treating of gastrointestinal infections and diarrhea. It also has antimicrobial properties. Therefore, several research studies have already been conducted to develop fermented and non-fermented products enriched with LGG, such as yoghurt, buttermilk, milk, fruit drinks, salad dressing, dry sausages, and cheese [22,23,24,25]. The aim of this study was to evaluate the viability of probiotic LGG in kefir products made with kefir grains and natural kefir starter cultures without grain. Moreover, quality characteristics namely organoleptic, syneresis, chemical, and rheological properties of kefir products from kefir grain and natural kefir starter culture with added LGG were compared to those of a control product over 21 days of storage at 4 °C.

## 2. Materials and Methods

### 2.1. Preparation of Kefir Samples

Kefir was manufactured according to the methods of Kök–Taş et al. [11] and Irigoyen et al. [26]. Commercial full-fat ultra-high temperature (UHT) milk (3.6 g/100 g of fat, 12.8 g/100 g of total solids (TS) content, and pH of 6.67) was used. Kefir grains (KG) were purchased from prebiotic company (Tehran, Iran). The grains were inoculated in UHT milk at room temperature and kept for short periods; the medium was replaced with fresh UHT milk daily to maintain the grains’ viability. The grains were activated to obtain high amounts of the kefir grain biomass. Kefir grains and natural kefir starter culture (KS) were used to ferment milk for kefir production. Natural kefir starter culture was obtained from kefir grains by straining the grains using a cloth sieve after fermentation at 28 °C for approximately 20 h; the final fermentation pH was 4.6. Fermentation time (20 h) was the same for KG and KS. The inoculation rate of the culture was selected after preliminary sensory studies. Briefly, a sensory panel of 12 semi-trained and experienced panelists tested kefir samples cultured with different inoculation rates (2, 3, and 5%), and evaluated them according to overall presentation (taste, smell, texture, and appearance). The optimal inoculation rates were 2% for KG (wt/vol) and 3% for KS (wt/vol). 

### 2.2. Manufacturing of Kefir Beverages

**Figure 1:**
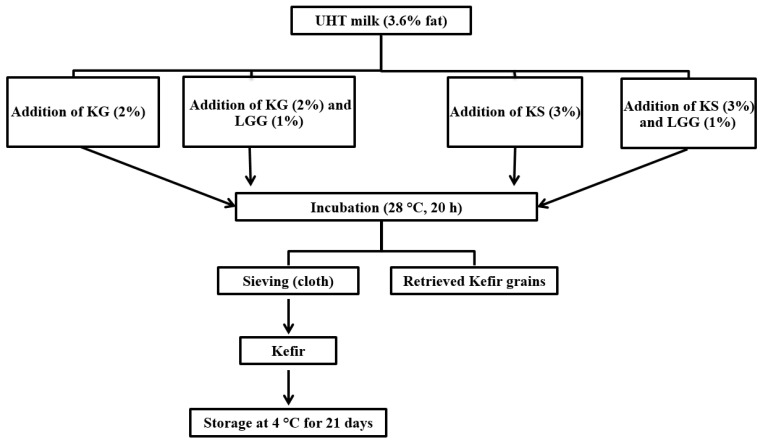
Manufacturing flowchart of different formulations of kefir. KG = kefir produced from kefir grains; KS = kefir produced from natural kefir starter culture; LGG = *Lacticaseibacillus rhamnosus* GG.

Before the kefir preparation, *Lacticaseibacillus rhamnosus* GG strain (LGG; ATCC 53103) [27] was cultured in de Man, Rogosa, Sharpe (MRS; Oxoid, Basingstok, Hampshire, UK) medium at 37 °C for 24 h under anaerobic conditions. Following incubation, 50 μL of probiotic culture was sub-cultured in plastic tubes containing 50 mL of MRS broth, which were incubated at 37 °C overnight under anaerobic conditions. Next, the probiotic culture biomass was harvested by centrifugation at 4000× *g* for 10 min at 20 °C, and washed twice with sterilized standard saline solution. Finally, the harvested cells were resuspended in 10 mL of UHT milk and used as a LGG culture to produce kefirs. Four different kefir batches were prepared: KG (kefir produced from kefir grains), KG-LGG (kefir made from kefir grains, with the addition of LGG), KS (kefir produced from natural kefir starter culture), and KS-LGG (kefir produced from natural kefir starter culture, with the addition of LGG). All batches were incubated at 28 °C for 20 h (Figure 1). Kefir samples containing the LGG strain were prepared using 1% probiotic LGG with 2% KG and 3% KS. All samples were filtered by a cloth sieve to filter out the grains. After kefir production, the samples were kept for 21 days at 4 °C. Probiotic viability and physicochemical properties were analyzed after 1, 7, 14, and 21 days of storage.

### 2.3. Physicochemical Analysis of Kefir: pH, Total Titratable Acidity (TTA), Syneresis

pH values of the kefir were determined using a pH meter (Thermo Orion Model-420A′). In addition, TTA was measured by the AOAC official method [28]. The syneresis values of the kefir samples were determined as recommended by Aryana [29]. Briefly, 100 g of each kefir batch was weighted on a fine mesh screen (14 µm) placed on top of a funnel. Syneresis is expressed as the amount of whey separated from the samples under the force of gravity at room temperature after 2 h of drainage into a flask of known weight, divided by the initial kefir mass. 

### 2.4. Enumeration of LGG

First, 1.0 mL of each sample was diluted with 9 mL of physiological saline solution. Samples were then serially diluted; using the spread-plate technique in agar made of modified MRS (mMRS (g L^−1^): peptone (Fisher Scientific, Janssen-Pharmaceuticalaan, Belgium), 10; yeast extract (Acros Organics, NJ, USA), 4; K_2_HPO_4_ (Merck, Darmstadt, Germany), 2; NaCH3CO_2_ · 3H_2_O (Oxoid, Hampshire, UK)), 5; triammonium citrate (Fisher Scientific, Loughborough, UK), 2; MgSO_4_ · 7H_2_O (J.T.Baker, Deventer, The Netherlands), 0.2; MnSO_4_ · H_2_O (Merck, Darmstadt, Germany), 0.05) supplemented with 1% (wt/vol) lactose and 50 µg/mL of bromocresol purple (BCP), the plates were incubated at 37 °C for 24–48 h in an anaerobic jar. During incubation, lactose-positive bacteria produce lactic acid by the fermentation of lactose, which lowers the pH of the media, changing the color of BCP from purple to yellow, whereas lactose negative bacteria grow as small colonies utilizing peptone and yeast extract for growth without production of lactic acid explaining why the colonies remain purple.

Since LGG does not utilize lactose, colonies without acid production did not change color; these were streaked onto MRS agar plates to obtain pure cultures for identification of LGG colonies by strain-specific PCR. For each amplification reaction, a positive control (*L. rhamnosus* GG strain (ATCC 53103) and a negative control (double distilled water) were included. Next, the lactose-negative colonies (presumptive LGG strains) were used as templates in colony PCR using primers specific to *L. rhamnosus* GG in Eppendorf Mastercycler (Hamburg, Germany) for the amplification of a fragment containing hypothetical conserved protein gene LGG_00154 using 5′-CGCCCTTAACAGCAGTCTTC-3′ and 5′-GCCCTCCGTATGCTTAAACC-3′ sequences as forward and reverse primers, respectively [27], yielding a 757-bp product. Phusion high-fidelity DNA polymerase (Thermo Fisher Scientific, Waltham, MA, USA) was used for PCR amplification. PCR was carried out in the final volume of 25 µL containing 20 pmol in each primer, Phusion HF buffer, 0.2 mM of deoxynucleotide triphosphates, 0.5 U of Phusion DNA polymerase, and cells from a single colony as the template under the following conditions: 30 s at 98 °C as an initial denaturation step, followed by 35 cycles of denaturation at 98 °C for 10 s, annealing at 60 °C for 30 s, and extension at 72 °C for 30 s, and a final extension step of 72 °C for 5 min. The amplification products (5 µL) were then separated by electrophoresis in 0.5 mg/mL ethidium bromide-stained 1% (wt/vol) agarose gel, and the DNA was visualized by a ChemiDoc MP imaging system (Bio-Rad, Hercules, CA, USA). The size markers used were 100-bp GeneRuler DNA ladders (Thermo Fisher Scientific). Colonies resulting in an amplified fragment of a size corresponding to the expected 757-bp fragment were regarded as LGG strains [30].

### 2.5. Sensory Analysis of Kefir

The assessment of flavor, body, texture, color, appearance, and overall acceptability of all kefir samples was performed by a panel of 15 semi-trained and experienced members (students, academic staff and faculty members at the University of Helsinki, Helsinki, Finland). Kefir samples were served to the panelists in transparent glass cups bearing 3-digit random codes. Each sample was scored individually on a 5-point hedonic scale ranging from 1 (dislike extremely) to 5 (like extremely) on the tenth day of storage. Evaluators were instructed to rinse their mouths with drinking water before tasting each sample.

### 2.6. Determination of Apparent Viscosity and Gel Structure of Kefir

The viscosities of the kefir samples were measured with a HAAKE MARS 40 rheometer (Thermo Scientific, Germany) with a parallel-plate (diameter 35 mm) measuring system. The flow behavior was determined by a rotational measurement in which the shear rate was first increased in a stepwise manner from 0.3 to 300 1/s (20 steps), then decreased from 300 to 0.3 1/s (20 steps). All viscosities were measured at 4 °C (the storage temperature of the kefir). The system was stabilized for 2 min before analysis. The apparent viscosities of the kefir were reported at a shear rate of 23.5 1/s when rotation shear rate increased from 0.3 to 300 1/s. Hysteresis loop area, which indicates the structural degradation, was reported by calculating the change in area of the flow profile between 0.3 to 300 1/s and 300 to 0.3 1/s, using RheoWin Measuring and Evaluation Software. To demonstrate the viscoelastic properties of the kefir during storage, a frequency sweep was conducted from 0.1 to 10 Hz using oscillatory measurement with a strain that was selected from the linear viscoelastic range. The G′ (storage modulus) and G″ (loss modulus) were reported at a frequency of 1.46 Hz. Two biological replicates were analyzed for each timepoint, and each replicate was measured twice for all tests.

### 2.7. Statistical Analysis

All physicochemical analyses and microbial enumerations were conducted in triplicates. The data obtained for kefir’s physicochemical, microbial, and organoleptic evaluation were analyzed with ANOVA using the General Linear Model procedure, reported as mean ± standard deviations. Tukey’s test was used to compare the means; significant differences were estimated based on a *p* < 0.05. All statistical analyses were carried out using Minitab 16 program (Minitab Inc., State College, PA, USA).

## 3. Results and Discussion

### 3.1. PH and TTA of Kefir

**Figure 2:**
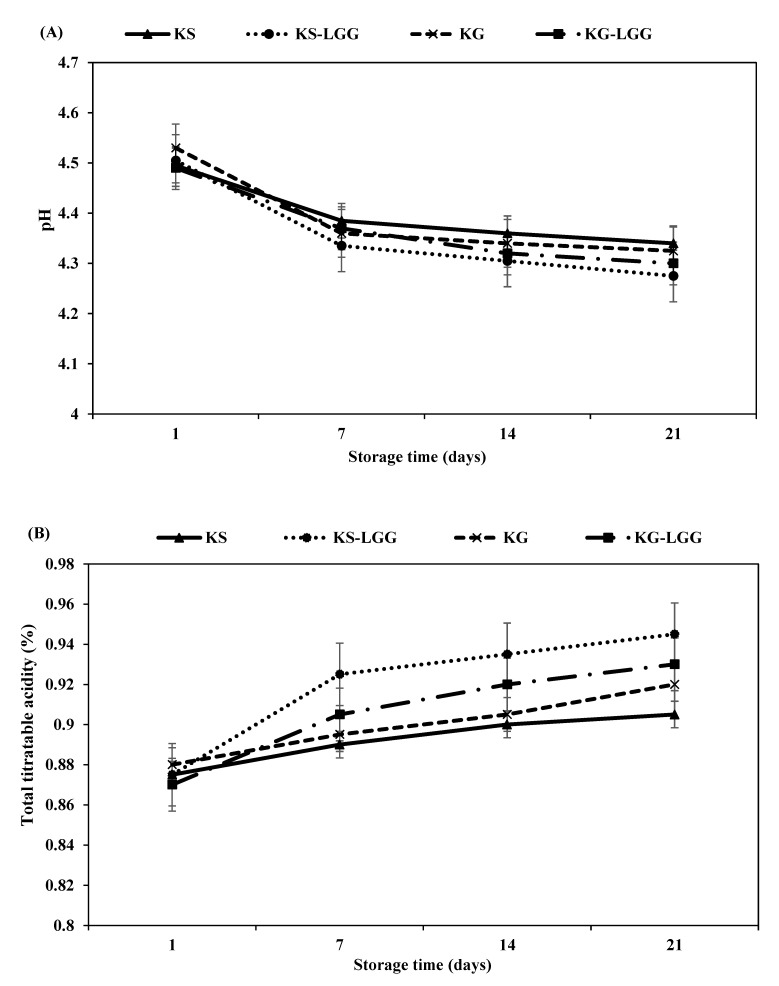
pH (**A**) and total titratable acidity (as lactic acid %) (**B**) of different formulations of kefir during storage at 4 °C. KG = kefir produced from kefir grains, KG-LGG = kefir produced from kefir grains containing LGG, KS = kefir produced from natural kefir starter culture, and KS-LGG = kefir produced from natural kefir starter culture containing LGG. Error bars represent the mean (*n* = 3) ± standard deviation (SD).

The pH values of the kefir samples were measured after 1, 7, 14, and 21 days of storage at 4 °C. The index of pH of all kefir samples ranged between 4.49 and 4.53 on day 1; these values decreased throughout the storage period, as also reported in other fermented milk products such as yoghurt [31,32]. pH values of KG varied from 4.53 to 4.32, and from 4.49 to 4.34 in KS throughout the duration of storage time (Figure 2A). These values were higher than those reported by Kök–Taş et al. [11], who noted pH values of 4.47–4.29 for KG, and 4.50–4.37 for KS during 21 days of storage at 4 °C.

The mean pH values were similar between kefir cultures with and without probiotic strain (*p* > 0.05) (Figure 2A), in accordance with results reported by Mitra and Ghosh [33], who also observed a slight but insignificant decrease in pH values in kefir samples containing LGG. During the storage period, pH values of the kefir samples made with natural kefir starter culture and the added probiotic LGG strain were significantly different (*p* < 0.05) from those without the added LGG probiotic. Notably, the pH of kefir products is correlated with their acidity [2,11,33]. Lactic acid is the most widespread acid produced by probiotic bacteria [31]. In this study, the lactic acid content (%) of KG and KS samples varied from 0.88 to 0.92, and from 0.87 to 0.90, respectively, during the 21 days of storage time (Figure 2B). In a similar study, Kök–Taş et al. [11] found the lactic acid content of KG and KS samples to range from 0.89 to 0.92, and from 0.83 to 0.89, respectively, during 21 days of storage at 4 °C. These slight differences may occur due to the yeast content limiting the propagation of LAB [34]. The acidity of the kefir grains was similar to that of the natural kefir starter culture with LGG probiotic strain (*p* > 0.05) (Figure 2B). The increased acidity in KG-LGG and KS-LGG compared to KG and KS may be assigned to metabolism of facultatively heterofermentative in LGG that transforms hexoses into L (+)-lactic acid through the Embden–Meyerhof pathway, as previously described [20].

### 3.2. Syneresis of Kefir

**Figure 3:**
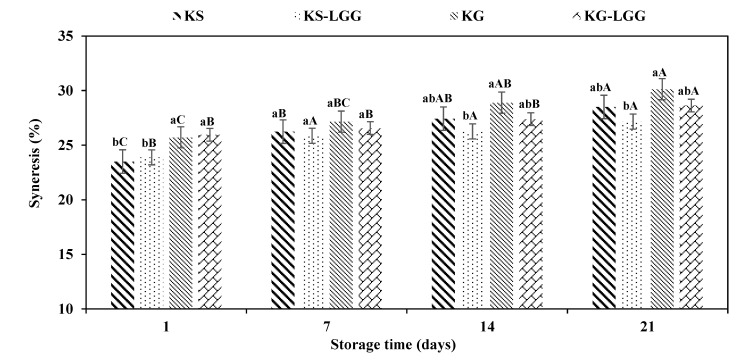
Syneresis (%) of different formulations of kefir during storage at 4 °C. KG = kefir produced from kefir grains, KG-LGG = kefir produced from kefir grains containing LGG, KS = kefir produced from natural kefir starter culture, and KS-LGG = kefir produced from natural kefir starter culture containing LGG. Lowercase letters indicate significant differences (*p* < 0.05) between samples at the same storage timepoint; uppercase letters indicate significant differences (*p* < 0.05) between the storage days of each kefir sample. Error bars represent the mean (*n* = 3) ± standard deviation (SD).

As shown in Figure 3, the syneresis of kefir samples increased during the 21 days of storage at 4 °C. Previous research has also shown an increase of syneresis in other fermented dairy products such as soymilk kefir and yoghurt during storage [35,36]. In this study, the syneresis of KG samples ranged from 25.71 to 30.12, and hence a greater whey separation than KS samples, whose syneresis values ranged from 23.50 to 28.50 at 7, 14, and 21 days at 4 °C (*p* > 0.05). These results are in agreement with Montanuci et al. [37], who reported greater syneresis of a formulation fermented with kefir grain compared to the formulation fermented with starter culture [37]. Our finding revealed that the syneresis of two formulations containing probiotic bacteria (KS-LGG and KG-LGG) was lower than the kefir samples made without LGG probiotic strain (KS and KG) at 7, 14, and 21 days of refrigerated storage (*p* > 0.05). However, the syneresis of KS-LGG and KG-LGG was higher than KS and KG, respectively, after the first storage day at 4 °C. One explanation for this result may be a decrease in pH during fermentation that enhances the resistance of casein particles to syneresis [38].

KS-LGG products showed a slight decrease in syneresis compared with KG-LGG, which may be associated with pH and lactic acid % [39,40]. Interestingly, we found that the syneresis values of KS-LGG and KG-LGG products showed significant differences only on day 1 of storage (*p* < 0.05). However, on days 7, 14, and 21 of the storage period, KS-LGG exhibited lower whey separation than KG-LGG. Several factors can contribute to this parameter, such as accumulation of organic acids, post-acidification [37], kefir concentration [41], kefiran concentration [42], total solid, and milk composition [43].

### 3.3. Viability of LGG during Kefir Storage

The survival of LGG bacteria in kefir beverages made with kefir grain as a carrier has already been reported [33]. However, no studies have confirmed this finding using natural kefir starter culture as a potential probiotic carrier in KS-LGG cocktail. It is generally demonstrated that the beneficial effect of probiotic bacteria in the gut environment depends on their population at a minimum level, which means that dairy products marketed as fermented probiotics should contain at least 10^7^ cfu/mL of viable probiotic cells [21,44].

**Figure 4:**
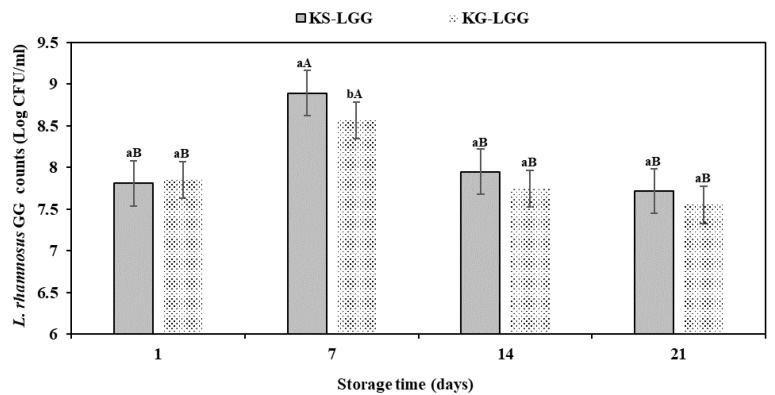
Viability of LGG in different formulations of kefir during storage at 4 °C. KG = kefir produced from kefir grains, KG-LGG = kefir produced from kefir grains containing LGG, KS = kefir produced from natural kefir starter culture, and KS-LGG = kefir produced from natural kefir starter culture containing LGG. Lowercase letters indicate significant differences (*p* < 0.05) between samples at the same storage timepoints; uppercase letters indicate significant differences (*p* < 0.05) between the storage days of each kefir sample. Error bars represent the mean (*n* = 3) ± standard deviation (SD).

In the current research, the viability of LGG bacteria in kefir grains and natural kefir starter culture was studied during a storage time of 21 days at 4 °C (Figure 4 and Supplementary Figure S1). After the first storage day at 4 °C, LGG counts of KS-LGG and KG-LGG samples were 7.80 and 7.85 log cfu/mL, respectively (Figure 4). It should be noted that the LGG counts in KS-LGG and KG-LGG steadily increased up to 7 days of storage. Accordingly, the highest LGG counts were observed in different formulations of KS-LGG and KG-LGG after one week. At this point, the LGG counts of KS-LGG samples were significantly higher than in the KG-LGG samples (8.89 and 8.56 log cfu/mL, respectively) (*p* < 0.05; Figure 4). These results agree with the LGG counts reported in the literature: LGG in combination with kefir grain increased to 7.45 log cfu/g until 6 days of storage [33]. Generally, kefir contains a diverse bacterial population; cell counts reportedly range between from 4.80 to 8.92 log cfu/mL [45]. Bacteria such as LAB and lactobacilli as proteolytic organisms are capable of degrading milk proteins into smaller peptides and amino acids [46]. It is commonly recognized that LGG bacteria do not effectively degrade β-casein, but can hydrolyze α_s1_-casein [27,47]. Hence, LGG bacteria can utilize casein-derived peptides and amino acids for growth [47]. In accordance with our LGG counts after 7 days of storage, [48] similarly reported that *L. rhamnosus* bacteria viability ranged between 8 and 9 log cfu/mL in casein hydrolysate and milk protein samples, respectively [48]. It was later shown that after 7 days of product fermentation at 4 °C, LGG viability was between 7 and 8 log cfu/mL in co-culture with *Streptococcus thermophilus* [49]. In contrast to our findings, [50] reported that the viability of LGG decreased from 6.88 to 6.70 log cfu/mL in a cocktail culture with *S. thermophilus*, *Lactobacillus acidophilus*, *Lactobacillus bulgaricus*, and *Bifidobacterium lactis* from days 1 to 7 at 4 °C [50].

After 7 days of storage, LGG counts gradually declined until 21 days of storage (*p* < 0.05), where the LGG final counts in KS-LGG and KG-LGG samples reached 7.71 and 7.55 log cfu/mL, respectively (Figure 4). On days 14 and 21 of storage, LGG counts were similar between KS-LGG and KG-LGG samples (*p* > 0.05). It is assumed that pH decline and post-acidification during storage of kefir samples (Figure 2) may lead to an adverse effect on the survival rate of probiotic bacteria [7,31].

### 3.4. Sensory Acceptance of Kefir

A high-quality kefir beverage should have a satisfying flavor and high-grade maintenance properties [6,51]. The scores collected for flavor, body and texture, color and appearance, and overall acceptability of kefir samples with and without LGG bacteria are presented in Table 1. The flavor score of KS samples was significantly higher (*p* < 0.05) than KS-LGG, KG, and KG-LGG samples, indicating that lower acidity and yeasty flavor in natural kefir starter culture was preferred by the evaluators (Table 1). The superior flavor of KS may likely be associated with lower acidity in comparison to the other kefir samples, which is evident from the pH and TTA parameters (Figure 2). On the other hand, yeasty flavor and higher acidity may cause the lower flavor score in the KS-LGG samples; KS-LGG and KG-LGG samples were evaluated to have the lowest flavor scores. In line with our findings, Mitra and Ghosh [33] reported a similar observation in kefir grains with an LGG-enriched formula. In another study, probiotic yoghurt samples containing *Lactobacillus reuteri* and *L. rhamnosus* bacteria also received lower flavor scores [52]. It should be noted that LGG bacteria can produce a variety of organic acids such as lactic acid, pyruvic acid, orotic acid, succinic acid, and uric acid that may affect the flavor score. Some researchers reported that the probiotic LGG bacteria produced pyruvic acid, orotic acid, succinic acid, and uric acid in probiotic fermented milk [53,54]. In the current study, organic acids produced by LGG bacteria likely affected flavor scores in KS-LGG and KG-LGG samples.

All the kefir formulations received similar scores for body and texture (*p* > 0.05), although these scores for KS-LGG and KG-LGG samples were lower than those for KS and KG samples. The differences in body and texture could be associated with lower amounts of exopolysaccharide produced by the probiotic LGG bacteria compared to kefiran’s biosynthesis by the microorganisms present in kefir [42,55]. As both the probiotic kefirs (KS-LGG and KG-LGG) appeared to have creamy consistency, and were both white and viscous, the color and appearance of the products did not differ significantly (*p* > 0.05). Notably, sieving the grains by cloth did not result in significant differences in body and texture scores. Similarly, there were no differences in color and appearance across the kefir formulations (*p* > 0.05). The same result was found for overall acceptability among the samples (*p* > 0.05). Several authors have investigated the effect of adding probiotic bacteria on sensory attributes in various fermented dairy products. For example, Ghaderi–Ghahfarokhi et al. [31] reported that the addition of *Lacticaseibacillus casei* strain into yoghurt did not produce any significant differences (*p* > 0.05) in appearance, body and texture, and overall acceptability compared to control samples. In another study, a camembert-type cheese enriched with *Lacticaseibacillus rhamnosus* GG was more acceptable than the control cheese, although these differences were not significant [56].

### 3.5. Rheological Characteristics of Kefir

Viscosity, hysteresis loop, and dynamic moduli indexes of kefir samples prepared with different formulations are presented in Table 1, Table 2 and Table 3, respectively. The hysteresis loop is considered as a reversible, isothermal, time-dependent decrease in the apparent viscosity when a material is subjected to increased shear rate [57]. The viscosity of the kefir samples ranged between 1197.1 ± 50.2 to 1269.6 ± 173.7 mPa·s on day 1 at 4 °C (Table 1). At first, the viscosity parameter seemed to be independent of fermentation, type of starter, and addition of probiotic bacteria. As shown in Table 2, KS samples had a higher viscosity than other kefir samples after the first day of storage. Similarly, Kök–Taş et al. [11] reported that the viscosity of kefir made with natural kefir starter culture was higher than that made with kefir grains on day 1 of cold storage. The decrease of viscosity with the increase of the shear rate suggests pseudoplastic behavior. One possibility is that sieving kefir grains after fermentation prohibited the formation of the gel network, which could affect the viscosity [37,58]. Similar observations were reported in other studies [11,26]. Unlike our findings, Mitra and Ghosh [33] reported a lower apparent viscosity of kefir prepared with starter compared to kefir produced by kefir grain. We found that unlike KS-LGG samples, the viscosity of KG-LGG samples was higher than that of the control kefir (KG) (*p* > 0.05) after day 1 of storage. 

As presented in Table 2, Table 3 and Table 4, the indexes of viscosity, hysteresis loop, and dynamic moduli were similar across the various formulations of kefir, indicating that kefir products were similar in appearance and mouth-feel regardless of whether they were made with kefir grain or starter culture. These levels remained the same during 21 days of storage. Moreover, the results showed that the viscosity of kefir products correlated well with their thixotropic behavior, which is a significant issue in hydrogel rheological properties. The return of the hydrogel to its initial structure, known as hysteresis loop, was evaluated by observing the viscosity change during the restoration process after shearing [59,60].

In this study, the viscosity of KS and KG samples gradually increased during storage at 4 °C, except for the decrease in viscosity in the KG sample at day 21 compared to the previous days of storage time. In agreement with our results, Beshkova et al. [61] reported that the viscosity of kefir made with pure culture and kefir grain increased after 7 days. Further, the hysteresis loop and viscosity of KS-LGG and KG-LGG samples were higher than KS and KG, respectively, at 7 days of storage. It is reasonable to assume that an interaction between milk proteins and the extracellular polysaccharide, kefiran, may influence viscosity. Some studies at the molecular level showed that EPS materials could bind water, and the interaction of proteins may be associated with increased viscosity and thixotropic behavior in these products [59,62].

In the current study, the hysteresis loop, storage modulus (G′), loss modulus (G″), and viscosity of KS-LGG samples were lower than KS samples, except at 7 days of storage. The storage modulus G′ > loss modulus G″ suggested gel-like structure, while loss tangent (tan δ) indicated the strength of the gel supported by the milk protein network and the production of exopolysaccharides. Combined with results from previous studies, these findings advance our understanding of the mechanisms of viscosity reduction by reduced EPS levels, which results from hydrolyzation of EPS into its monomers by glycohydrolases [63,64]. It has been reported [65] that the viscosity of EPS production by *L. rhamnosus* was reduced by glycohydrolases during cold storage. In this study, both storage (G′) and loss (G″) modulus of KS-LGG were lower than KS during storage (Table 4). This decrease can be attributed to the hydrolyzation of EPS due to glycohydrolases and increased acidity (Figure 2) as compared with control kefir (KS) [66,67]. In other research, EPS produced by bacteria was recognized to interact with milk proteins and improve viscoelastic properties of weak gels such as yoghurt [68]. Therefore, we could expect that the decrease in EPS level may have reduced the viscosity values of the KS-LGG samples in our study. The reduced viscosity of KS-LGG compared to the control kefir (KS) could be related to sensory acceptance, as the evaluators voted for kefir with slightly thicker composition, which may result from more negligible EPS and increased acidity.

## 4. Conclusions

We manufactured kefir products from a variety of starting products, including kefir grains and natural kefir starter culture with and without probiotic LGG bacteria. LGG-enriched natural kefir starter culture showed an average LGG count of 8.09 log cfu/mL during 21 days of storage at 4 °C. In addition to this benefit, in sensory analysis at 10 days of storage, although natural kefir starter culture achieved the highest overall acceptability score close to the maximum, other kefir products were evaluated as having satisfactory sensorial acceptance. Based on the rheological and the probiotic LGG bacteria viability observations during a period of refrigerated storage, we postulated that kefir with an LGG-enriched formula could potentially be used as a beverage, since the addition of LGG bacteria to natural kefir starter culture did not significantly affect the viscosity properties. Taken together, using natural kefir starter culture without grains is more convenient and applicable than retaining kefir grains in large-scale industrial production. As such, probiotic LGG bacteria could be a potential alternative to produce kefir with remedial efficacy in the dairy industry. However, further studies should be conducted to evaluate the microbial analysis and organic acid profile in kefir grain and natural kefir starter culture.

## Figures and Tables

**Table 1:** Sensory scores of kefirs with different formulations.

Kefir Formulations ^1^	Sensory Attributes			
	Flavor	Body and Texture	Color and Appearance	Overall Acceptability
KS	4.46 ± 0.63 ^a^	4.20 ± 0.84 ^a^	4.00 ± 0.84 ^a^	4.26 ± 0.45 ^a^
KS-LGG	3.20 ± 0.67 ^b^	3.93 ± 1.09 ^a^	4.33 ± 0.61 ^a^	3.80 ± 0.86 ^a^
KG	3.60 ± 0.82 ^b^	4.26 ± 0.70 ^a^	4.40 ± 0.84 ^a^	3.86 ± 0.51 ^a^
KG-LGG	3.20 ± 0.73 ^b^	4.06 ± 0.79 ^a^	4.00 ± 0.77 ^a^	4.06 ± 0.70 ^a^

^a–b^ Values (average ± SD) in the same column with the same lowercase letter are not significantly different (*p* > 0.05). ^1^ Abbreviations of different kefir formulations: KG = kefir produced from kefir grains, KG-LGG = kefir produced from kefir grains containing LGG, KS = kefir produced from natural kefir starter culture, and KS-LGG = kefir produced from natural kefir starter culture co containing LGG.

**Table 2:** Viscosity (mPa·s) of kefir samples at 23.5 1/s shear rate.

Kefir Formulations ^1^	Storage Period (d)			
	1	7	14	21
KS	1269.6 ± 173.7 ^a,A^	1300.0 ± 0.0 ^a,A^	1340.0 ± 28.3 ^a,A^	1345.0 ± 7.1 ^a,A^
KS-LGG	1225.8 ± 6.2 ^a,A^	1315.0 ± 7.1 ^a,A^	1175.0 ± 120.2 ^a,A^	1250.0 ± 56.6 ^a,A^
KG	1197.1 ± 50.2 ^a,A^	1225.0 ± 77.8 ^a,A^	1240.0 ± 56.6 ^a,A^	1200.0 ± 0.0 ^a,A^
KG-LGG	1262.9 ± 25.5 ^a,A^	1360 ± 56.6 ^a,A^	1275 ± 49.5 ^a,A^	1260 ± 14.4 ^a,A^

^a^ Values (average ± SD) in the same column with the same superscript letters are not significantly different (*p* > 0.05). ^A^ Values (average ± SD) in the same row with the same superscript letters are not significantly different (*p* > 0.05) between the storage days of each kefir sample. ^1^ Abbreviations of different kefir formulations: KG = kefir produced from kefir grains, KG-LGG = kefir produced from kefir grains containing LGG, KS = kefir produced from natural kefir starter culture, and KS-LGG = kefir produced from natural kefir starter culture containing LGG (*n* = 2).

**Table 3:** Hysteresis loop area Pa/s of kefir samples between upper and lower area of the flow curves.

Kefir Formulations ^1^	Storage Period (d)			
	1	7	14	21
KS	3094.5 ± 481.5 ^a,A^	3116.5 ± 30.4 ^a,A^	3067.5 ± 152.0 ^a,A^	3075.5 ± 0.7 ^a,A^
KS-LGG	3010 ± 42.4 ^a,A^	3160.5 ± 29.0 ^a,A^	2667 ± 272.9 ^a,A^	2834 ± 183.8 ^a,A^
KG	2646 ± 152.7 ^a,A^	2592.5 ± 191.6 ^a,A^	2657 ± 161.2 ^a,A^	2520 ± 19.8 ^a,A^
KG-LGG	2975.5 ± 40.3 ^a,A^	3186.5 ± 180.3 ^a,A^	2925 ± 100.4 ^a,A^	2837.5 ± 57.3 ^a,A^

^a^ Values (average ± SD) in the same column with the same superscript letters are not significantly different (*p* > 0.05). ^A^ Values (average ± SD) in the same row with the same superscript letters are not significantly different (*p* > 0.05) between the storage days of each kefir sample. ^1^ Abbreviations of different kefir formulations: KG = kefir produced from kefir grains, KG-LGG = kefir produced from kefir grains containing LGG, KS = kefir produced from natural kefir starter culture, and KS-LGG = kefir produced from natural kefir starter culture containing LGG (*n* = 2).

**Table 4:** Storage modulus G′, loss modulus G″, and loss tangent (tanδ  = G″/G′) of kefir samples at frequency of 1.46 Hz.

Kefir Formulations ^1^	Storage Period (d)											
	d 1			d 7			d 14			d 21		
	G′	G″	tan(δ)	G′	G″	tan(δ)	G′	G″	tan(δ)	G′	G″	tan(δ)
KS	200.6 ± 73.4 ^a,A^	53.5 ± 20.0 ^a,A^	0.266 ± 0.002 ^a,A^	177.4 ± 5.7 ^a,A^	44.2 ± 1.0 ^a,A^	0.249 ± 0.019 ^a,A^	181.8 ± 1.27 ^a,A^	46.2 ± 2.6 ^a,A^	0.254 ± 0.012 ^a,A^	190.3 ± 24.0 ^a,A^	46.4 ±5.6 ^a,A^	0.244 ± 0.001 ^a,A^
KS-LGG	156.8 ± 12.2 ^a,A^	42.3 ± 1.80 ^a,A^	0.270 ± 0.009 ^a,A^	176.5 ± 17.3 ^a,A^	46.2 ± 1.6 ^a,A^	0.262 ± 0.016 ^a,A^	171.1 ± 17.8 ^a,A^	43.4 ± 1.5 ^a,A^	0.254 ± 0.017 ^a,A^	160.3 ± 9.0 ^a,A^	42.3 ± 1.3 ^a,A^	0.253 ± 0.005 ^a,A^
KG	146.6 ± 0.1 ^a,A^	40.3 ± 3.1 ^a,A^	0.274 ± 0.021 ^a,A^	186.9 ± 31.3 ^a,A^	46.8 ± 8.5 ^a,A^	0.250 ± 0.003 ^a,A^	153.0 ± 28.2 ^a,A^	38.3 ± 7.8 ^a,A^	0.249 ± 0.005 ^a,A^	119.3 ± 7.4 ^a,A^	30.1 ± 0.7 ^a,A^	0.252 ± 0.009 ^a,A^
KG-LGG	203.8 ± 2.3 ^a,A^	53.7 ± 0.1 ^a,A^	0.263 ± 0.002 ^a,A^	185.5 ± 16.9 ^a,A^	46.0 ± 4.3 ^a,A^	0.248 ± 0.009 ^a,A^	143.1 ± 11.0 ^a,A^	35.2 ± 3.7 ^a,A^	0.246 ± 0.007 ^a,A^	153.9 ± 26.2 ^a,A^	36.9 ± 0.0 ^a,A^	0.239 ± 0.003 ^a,A^

^a^ Values (average ± SD) in the same column with the same superscript letters are not significantly different (*p* > 0.05). ^A^ Values (average ± SD) in the same row with the same superscript letters are not significantly different (*p* > 0.05) between the storage days of each kefir sample. ^1^ Abbreviations of different kefir formulations: KG = kefir produced from kefir grains, KG-LGG = kefir produced from kefir grains containing LGG, KS = kefir produced from natural kefir starter culture, and KS-LGG = kefir produced from natural kefir starter culture containing LGG (*n* = 2).

## Data Availability

All data related to the research are presented in the article.

## Supplementary Materials

The following supporting information can be downloaded at: www.mdpi.com/2304-8158/11/4/523/s1, Figure S1: Amplification of *Lacticaseibacillus rhamnosus* GG DNA. Lane M is a 100-bp ladder. Lanes 1,2,6, represent positive samples. Lanes 3,4 represent negative samples. Lane 5 is a positive control with *Lacticaseibacillus rhamnosus* GG (ATCC 53103) DNA. Lane 7 is a negative control without DNA.
