# Supplementary material for: Lacticaseibacillus rhamnosus GG Survival and Quality Parameters in Kefir Produced from Kefir Grains and Natural Kefir Starter Culture
Source: Foods. 2022 Feb 11;11(4):523. doi: 10.3390/foods11040523 (PMC8871425; doi:10.3390/foods11040523)
Supplement: Supplementary file 1 [file foods-11-00523-s001.zip › foods-1574034-supplementary-done.pdf]

foods

MDPI

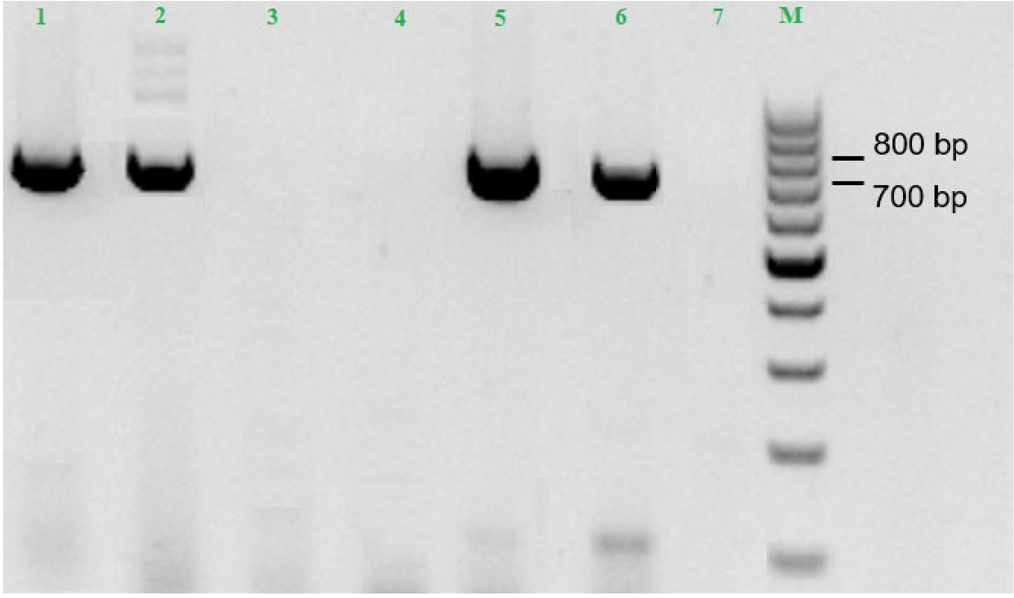

Figure S1: Amplification of *Lacticaseibacillus rhamnosus* GG DNA. Lane M is a 100-bp ladder. Lanes 1,2,6, represent positive samples. Lanes 3,4 represent negative samples. Lane 5 is a positive control with *Lacticaseibacillus rhamnosus* GG (ATCC 53103) DNA. Lane 7 is a negative control without DNA.
